# Biochemistry, Synthesis, and Applications of Bacterial Cellulose: A Review

**DOI:** 10.3389/fbioe.2022.780409

**Published:** 2022-03-08

**Authors:** Snehasish Mishra, Puneet Kumar Singh, Ritesh Pattnaik, Subrat Kumar, Sanjay Kumar Ojha, Haragobinda Srichandan, Pankaj Kumar Parhi, Rajesh Kumar Jyothi, Prakash Kumar Sarangi

**Affiliations:** ^1^ BDTC, Bioenergy Lab, School of Biotechnology, KIIT Deemed University, Bhubaneswar, India; ^2^ School of Biotechnology, KIIT Deemed University, Bhubaneswar, India; ^3^ Professor Brien Holden Eye Research Centre, LV Prasad Eye Institute, Hyderabad, India; ^4^ Department of Chemistry, Fakir Mohan University, Balasore, India; ^5^ Convergence Research Center for Development of Mineral Resources (DMR), Korea Institute of Geosciences and Mineral Resources (KIGAM), Daejeon, Korea; ^6^ Directorate of Research, Central Agricultural University, Imphal, India

**Keywords:** nanomaterials, cellulose fibrils, nanocellulose, cellulose biosynthesis, bacterial cellulose, bacterial nanocellulose, lignocellulosic biorefineries

## Abstract

The potential of cellulose nanocomposites in the new-generation super-performing nanomaterials is huge, primarily in medical and environment sectors, and secondarily in food, paper, and cosmetic sectors. Despite substantial illumination on the molecular aspects of cellulose synthesis, various process features, namely, cellular export of the nascent polysaccharide chain and arrangement of cellulose fibrils into a quasi-crystalline configuration, remain obscure. To unleash its full potential, current knowledge on nanocellulose dispersion and disintegration of the fibrillar network and the organic/polymer chemistry needs expansion. Bacterial cellulose biosynthesis mechanism for scaled-up production, namely, the kinetics, pathogenicity, production cost, and product quality/consistency remain poorly understood. The bottom-up bacterial cellulose synthesis approach makes it an interesting area for still wider and promising high-end applications, primarily due to the nanosynthesis mechanism involved and the purity of the cellulose. This study attempts to identify the knowledge gap and potential wider applications of bacterial cellulose and bacterial nanocellulose. This review also highlights the manufacture of bacterial cellulose through low-cost substrates, that is, mainly waste from brewing, agriculture, food, and sugar industries as well as textile, lignocellulosic biorefineries, and pulp mills.

## Introduction

The most abundant natural polymer available on Earth is cellulose (Morgan and Strumillo 2013), having tremendous economic importance across the globe. The primary end product of photosynthetic activity in both aquatic and terrestrial ecosystems is the cellulose exceeding to more than 100 billion tonnes per year by dry weight (Neo and Yang 2015), and is considered the most dominant renewable bioresource in the biosphere. The main industrial source of cellulose is dependent on multicellular plants such as the hard- and soft-wood trees, cotton, flax, jute, ramie, and hemp. Cellulose is a linear polysaccharide composed of glucose monomers, bound together through condensation polymerization reactions of long chains of anhydro-glucose units by β-1,4-glycosidic linkage. Cellulose is synthesized by diverse members from the kingdoms Plantae and Animalia and domain *Eubacteria* (Abeer, 2014; McNamara et al., 2015). Among all, plant cell walls represent the major source of cellulose, although such types are usually associated with lignin, pectin, and hemicellulose fractions. Apart from plants, a number of distinct and specialized bacterial, algal, and slime mold groups, particularly inhabiting the soil environment predominantly rich in organic matter, also synthesize cellulose. The synthesis of cellulose has been observed in *Dictyostelium* (a multicelled eukaryotic, phagotrophic bacterivore) and tunicates (marine chordate) from the animal kingdom (Abeer, 2014).

Cellulose, being a major constituent of the carbon cycle, is also a favorite among microbes and higher organisms, including cattle-carrying cellulolytic bacteria in the rumen on the one hand, and biotechnologists investigating the ways-and-means to convert cellulose into useful compounds on the other. Although the molecular mechanisms of cellulose synthesis have been elucidated successfully recently, many aspects yet remain unclear, specifically the mechanisms of cellular export of the nascent polysaccharide chain and organization of cellulose fibrils as the quasi-crystalline structure.

Naturally, cellulose is found in both pure (comprising about 35–50%) and complex (comprising about 50–65%) states on dry weight basis, with the complex state being associated primarily with the hemicelluloses and lignin at 20–35% and 5–30%, respectively (Behera et al., 2017). Physical properties like crystalline state, degree of crystallinity, and molecular weight of cellulose vary considerably, depending on its primary source. Being insoluble, diverse microbial (bacterial and fungal) groups degrade the cellulose extracellularly, providing carbon and energy sources to the associated microbial community in an ecological niche (Ojha et al., 2012).

**FIGURE 1:**
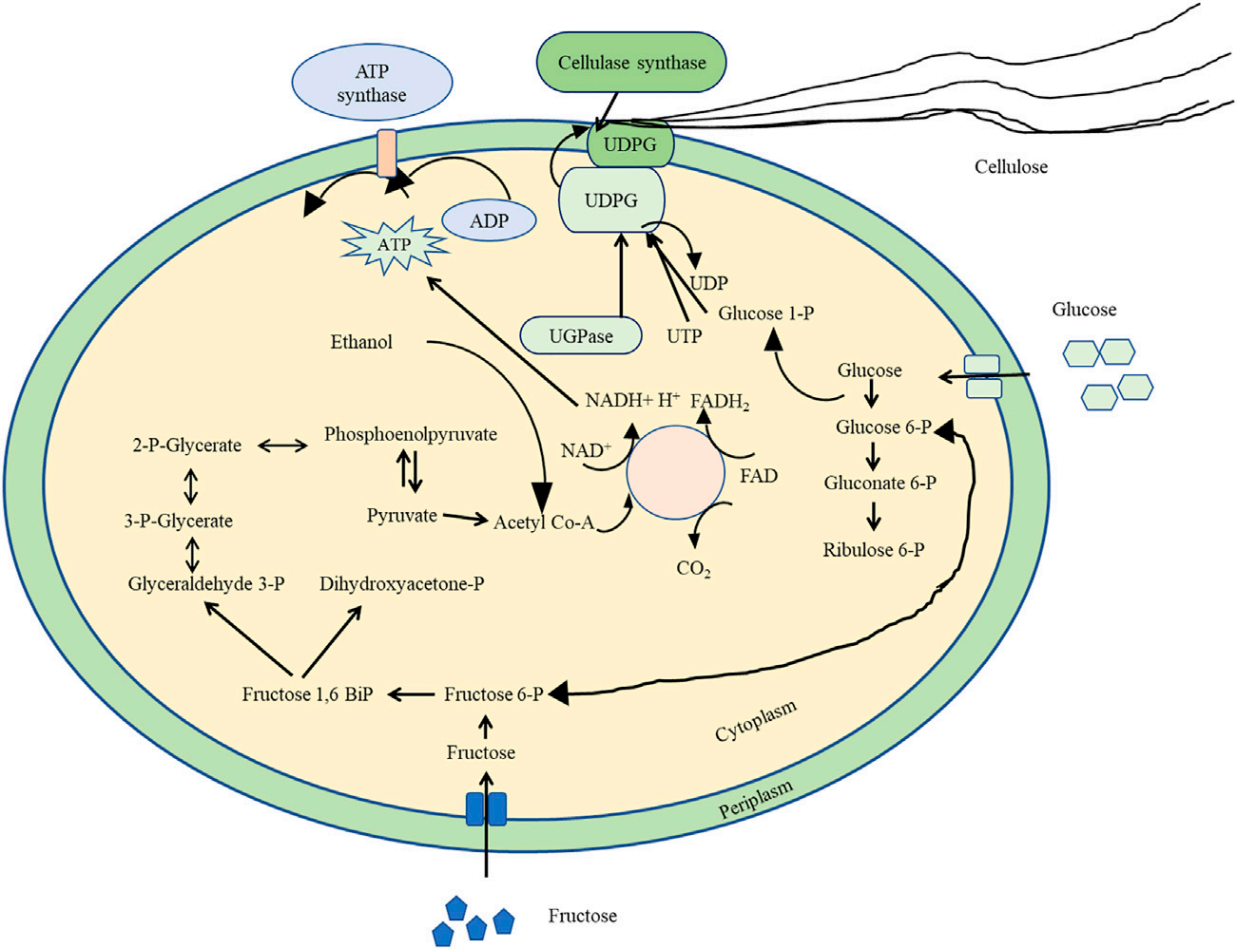
A detailed biosynthesis pathway of various biomolecules involved in bacterial cellulose synthesis of *K. xylinus*.

Most research on cellulose these days has been performed on either extraction of plant cellulose or synthesis of bacterial cellulose (BC) (Wang et al., 2016). Studies indicate that *Rhizobium*, *Azotobacter*, *Agrobacterium*, *Salmonella*, *Aerobacter*, *Acetobacter*, *Achromobacter*, and *Escherichia* sp. are the prominent genera involved in BC synthesis and/or metabolism (Seo et al., 2014; Ullah et al., 2017; Islam et al., 2021). BC synthesis in N_2_-fixier *Rhizobium leguminosarum*, *Burkholderia* sp., and *Pseudomonas putida* is well documented. Compared to plant cellulose, BC is a relatively pure cellulose form possessing certain unique and superior structural, physicochemical, and mechanical properties (Khattak et al., 2014 and Trache et al., 2016). Although plant cellulosic biomass is an abundantly occurring biopolymer, exploiting plant biomass for energy is difficult as its glycan polymer is recalcitrant to environmental biotransformation, paving the way to exploit microbial cellulose for their effective applications (Daniel et al., 2012; Basen et al., 2014; Mishra et al., 2018). Studies highlight about the selected microbial genera that can synthesize cellulose, yet species in particular such as *Gluconacetobacter xylinus*, a Gram-negative microorganism, have gained attention with regard to secreting cellulose as microfibrils in relatively higher quantity from a row of cellular synthetic sites (Stone et al., 2018; Volova et al., 2018). The microfibrils from each synthetic site combine in the external growth medium and form a large ribbon of cellulose. A floating pellicle-like structure is formed by the entrapment between cellulosic ribbons and the allied bacterial cells which lets these non-motile, strictly aerobic bacteria to grow in increased oxygen tension at the surface of the growth medium. *Agrobacterium tumefaciens*, a tumor-forming bacterium, excretes cellulose fibrils upon contact with the host plant cell that aid in cell attachment promoting its virulence (Heindl et al., 2014 and Park et al., 2015). *Gluconacetobacter xylinus* and *Agrobacterium tumefaciens* could be suitable candidate cellulose synthesizers because the genes, enzymes, and biosynthesis involved in the process have been found in them (Figure 1). Algal cellulose has been analyzed with regard to the crystalline features such as allomorphism (Park et al., 2015). In similar lines, the cellulosic algae proved useful for freeze-fracture studies where putative synthase complexes were first visualized in the plasma membrane, but without much success on the biochemical and molecular mechanisms (Schuurmans et al., 2014). The chordate *Dictyostelium discoideum* synthesizes cellulose at different stages in the life cycle, thus outlining defined cellulose synthase activity, as well as sophisticated genetic approaches for cellulose biosynthesis studies (Hobdey et al., 2016). The water mold of *Saprolegnia* comprises 1,4-β-D-glucan as well as β-D-1,3-glucan in its cell wall. Although different enzymes for glucan production *in vitro* have been described, genetic investigations show that this organism (Li et al., 2014) does not show any such developments from the epidermal layer of the ovule, and that they elongate synchronously within the boll. Yet no such experimental evidence has been reported, explaining that BC biosynthesis differs from that of plants, but uridine diphosphoglucose (UDPG) is highlighted as a vital intermediate in BC synthesis instead of guanidine diphosphoglucose (GDPG), which is associated in the biosynthesis in plants. Of the bacterial isolates *Gluconacetobacte*r sp. RV28, *Enterobacter* sp. RV11, and *Pseudomonas* sp. RV14 from rotten fruits and vegetables characterized through morphological and biochemical analysis, the mechanism of cellulose biogenesis in *Gluconacetobacter xylinus* that is capable of producing pure cellulose as an extracellular product was established. Furthermore, numerous process optimization for parameters such as inoculum density, temperature, pH, agitation, and carbon and nitrogen sources for growing this organism have been reportedly successful (Salehizadeh et al., 2018). In this review, a complete description on the recent developments on biochemistry, synthesis, and applications of BC has been discussed.

## Non-Bacterial Cellulose

The plant cell wall, the major non-bacterial source of cellulose, is mainly composed of two phases, matrix and microfibrils. Matrix is composed of polysaccharides like pectin, hemicellulose, phenolics, and protein, whereas microfibril is made up of a high degree of crystalline cellulose. Two special groups of cellulose synthases (CesA) are involved in both primary and secondary cell wall syntheses. Several fungi (particularly oomycetes) synthesize cellulose to enhance their pathogenicity. *Phytophthora infestans* (an oomycete) causing late blight in potato has a cell wall that is mainly composed of cellulose. Although four cellulose synthase genes (cesA) in *Phytophthora infestans* have been identified, its cellulose synthesis mechanism is yet unknown (Hua et al., 2015). Recently, a new solid nanocellulose fiber in marine seaweed named *Posidonia oceanica* was reported (Bettaieb et al., 2015). Marine biomass requires a simple medium to grow in controlled environment (Kim et al., 2015). Also, isolating cellulose from these marine weeds has less physiological barrier unlike plant biomass; no harsh chemical is required during down streaming. Although commercial production of this is still limited, some macroalgae such as *Valonia*, *Cladophora*, and *Boergesenia* are reportedly good cellulose nanofibril producers (Saito et al., 2013).

## Bacterial Cellulose Synthesis

As indicated earlier, bacterial genera like *Rhizobium* (*R. leguminosarum*), *Azotobacter*, *Agrobacterium*, *Salmonella*, *Aerobacter*, *Acetobacter*, *Achromobacter*, *Burkholderia*, *Pseudomonas putida*, and *Escherichia* sp. are some well-documented cellulose synthesizers/metabolizers. Table 1 lists some popular BC-producing bacterial strains. BC production was reported in both synthetic and non-synthetic media by *Gluconacetobacter*, *Agrobacterium*, and *Sarcina* through oxidative fermentation (Huang et al., 2014). The Gram-negative *Gluconacetobacter* is a major BC producer. After the initiation of BC synthesis, the glucose chains formed inside the bacterial cell leach out through the microscopic cellular pore and aggregate to create microfibrils, which leads to cellulose strip formation (Costa et al., 2014). These cellulose strips develop a web-like network, creating a highly porous matrix network. The cellulosic chain is held together with the help of hydrogen bonds. When used in the form of biocomposites, these BC fibrils are about 100 times smaller than plant cellulosic fibrils. Of the several bacterial species, *Acetobacter* sp. is the one known to produce cellulose in commercially viable quantity. The many products developed from BC include wound dressings (Chang and Chen 2016), high-quality additives to paper (Julkapli and Bagheri 2015), fiber glass filter sheets (Kamal et al., 2017), chewing gum (de Castro et al., 2017), food stabilizers (Paximada et al., 2016), and acoustic diaphragms for audio instruments (Mango et al., 2016). Eventually, the wide availability of BC will undoubtedly lead to new applications and inventions.

**TABLE 1:** Bacterial cellulose production by some reported bacterial strains.

Sl. No	Bacterial strain	Supplement	Carbon source	Duration	Yield (g/L)	References
1	*Acetobacter xylinum* BRC 5	Oxygen, ethanol	Glucose	50 h	15.30	Halib et al. (2012)
2	*Acetobacter xylinum* BPR2001	Oxygen, agar	Fructose	72 h	14.10	Shi et al. (2014)
3	*Acetobacter xylinum* BPR2001	—	Molasses	72 h	7.82	Phisalaphong and Chiaoprakobkij (2012)
4	*Acetobacter xylinum* BPR2001	Agar	Fructose	56 h	12.00	Han et al. (2018)
5	*A. xylinum* ssp. sucrofermentans BPR2001	Oxygen, agar	Fructose	44 h	8.70	Lin et al. (2011)
6	*A. xylinum* ssp. sucrofermentans BPR2001	Oxygen	Fructose	52 h	10.40	Khirrudin (2012)
7	*Acetobacter xylinum* NUST4.1	Sodium alginate	Glucose	5 days	6.00	Xiao et al. (2012)
8	*Gluconacetobacter xylinus* IFO 13773	—	Molasses	7 days	5.76	Keshk and Sameshima, 2006
9	*Gluconacetobacter* sp. RKY5	—	Glycerol	144 h	5.63	Khirrudin (2012)
10	*Gluconacetobacter xylinus* strain K3	Green tea	Mannitol	7 days	3.34	Wu et al. (2013)
11	*Gluconacetobacter xylinus* IFO 13773	Lignosulfonate	Glucose	7 days	10.10	Karim et al. (2016)
12	*Acetobacter xylinum* E25	—	Glucose	7 days	3.50	Urbina et al. (2018)
13	*Acetobacter* sp. A9	Ethanol	Glucose	8 days	15.20	Shi et al. (2014)
14	*Acetobacter* sp. V9	Ethanol	Glucose	8 days	4.16	Hussain et al. (2017)
15	*Gluconacetobacter hansenii* PJK	Ethanol	Glucose	72 h	2.50	Hussain et al. (2017)
16	*Gluconacetobacter hansenii* PJK	Oxygen	Glucose	48 h	1.72	Lin et al. (2011)

The fibrous arrangements provide porosity and mechanical strength to BC (Esa et al., 2014). BC forms a white, thick, leathery structure in water–air contact. The molecular structural arrangement of BC is the same as phytocellulose, differing greatly in their physico-biological properties such as biocompatibility, porosity, purity, polymerization, tensile strength, water holding capacity, and reusability. (Wu et al., 2014).

## Advantage of Bacterial Cellulose

BC is a basic fibrillar structure of (C_6_H_10_O_5_)_n_ consisting of β-1→4 glucan chain. Glucan chains are held together by inter- and intra-hydrogen bondings (Ul-Islam et al., 2013). BC microfibrils are significantly smaller (about 100 times) than plant cellulose (Ahmed et al., 2017; Rastogi and Banerjee 2019). The BC networks of well-arranged 3-D nanofibers form a hydrogel sheet which poses high surface area and porosity. During its synthesis, protofibrils of glucose chain secrete through the bacterial cell wall and aggregation takes place between protofibrils for forming cellulose nanofibril ribbons (Stone et al., 2018). These BC ribbons construct the web network with a highly porous matrix (Panaitescu et al., 2016). Therefore, formed cellulose contains a substantial amount of surface hydroxyl groups, which explain its hydrophilicity, biodegradability, and chemical-modifying capacity (Heinze et al., 2018).

Since a long time, BC biosynthesis is being used as a simpler and genetically tractable model to analyze similar biosynthesis in plants. Even after this model system became non-essential, studies on BC biosynthesis provide useful insights, namely, polysaccharide export, regulation of bacterial cell responses to oxygen and nitric oxide (NO), cell motility, cell–cell interactions, biofilm formation and dispersal, and a variety of other environmental challenges, thus proving extremely important in their own right. Cellulose and its derivatives are accounted for a significant portion of extracellular matrix of biofilms and play important roles in restrain of virulence of important plant and human pathogens (Silva et al., 2016). Various approaches have been used to investigate BC synthesis (Ullah et al., 2016). By correlating the genome sequence with its biochemical and physiological information, it is now possible to reconstruct complete metabolic pathways. Although significant efforts have been made to enhance the *in vitro* synthesis of BC (Guerriero et al., 2015), a major challenge is to understand the cellular metabolism of BC at system level in this postgenomic era.

## Production of Bacterial Cellulose From Organic Waste

Significant worldwide economic, environmental, and energy challenges have heightened the relevance of long-term industrial waste management in the last decade. Because of advances of biotechnological techniques, scientists and researchers may now use renewable natural sources such as industrial wastes to create cellulosic polymeric materials like BC. Waste management and environmental cleanup will benefit from the use of these wastes for BC manufacturing, as well as the cost of waste disposal for enterprises. The industrial wastes utilized in BC production can be separated into numerous groups using this novel approach.

Agricultural waste is valued for its cultural and economic value around the world. It is a promising renewable energy source because of its ecologically favorable character, ease of availability, low cost, and long-term viability. Agricultural industries produce tonnes of biomass every day, but only 10% of it is employed as an alternative raw material in industries such as biomedical and automotive component manufacturing (Dungani et al., 2016). The use of agro-biomass (fig fruits, molasses, and palm date fruits) for BC manufacturing ultimately reduces costs, making it more sustainable, environmental-friendly, and marketable. Most agroindustries discard or reject coconut and pineapple juices as trash, but they are high in carbs and trace elements (Abol-Fotouh et al., 2020). These juices were tested as a medium for BC production, with coconut juice producing significantly more BC than pineapple juice (Islam et al., 2017). Endo-1, 4-xylanase, endo-1, 3(4)-glucanase, -amylase, subtilisin, and polygalacturonase were used to pretreat rice bark from agricultural leftovers. The BC generation of this enzymatic hydrolyzate, which contained up to 40 g/L of glucose, was tested. For aerated and static settings, BC production was found to be 1.57 g/L and 2.42 g/L, respectively. Orange peels were pretreated with cellulase and pectinase in order to raise the fermentable sugar concentration (60–80 g/L) so that they could be used as a substrate for BC synthesis (Islam et al., 2020). The results showed that utilizing pretreated orange peel medium produced 4.2–6.32 times more BC than while using standard medium (HS). Furthermore, structural research revealed that BC made from various wastes had thicker nanofibrils, despite no significant variations in the Fourier-transform infrared spectra (Kuo et al., 2017). In traditional rice wine distilleries, a large amount of makgeolli (a traditional Korean alcoholic beverage made up of rice) sludge is dumped. The makgeolli sludge medium has been reported to act as a carbon source for the growth of *G. xylinus* in the synthesis of BC. To encourage microbial growth, it contains glucose (10.24 g/L), organic acids (1.15 g/L), alcohol (0.93 percent v/v), total nitrogen (0.81 g/L), and metal ions. BC made from sugarcane molasses was shown to be non-cytotoxic, non-genotoxic, and non-acutely toxic, indicating its promise as a viable biomaterial for a variety of biological and medicinal applications (Pinto et al., 2016). Since the natural sources used in the fiber/textile sector are often high in cellulose content, the wastes created can be used to make various value-added products such as BC, followed by detoxification and hydrolysis treatments (Hussain et al., 2019).

## Molecular Biology of Microbial Cellulose Synthesis

A very promising line of genetic research of BC is directed toward obtaining industrially valuable strains by direct genetic manipulation of the genes encoding the cellulose synthesis catalysts, their adjunctive regulatory enzymes, and the relevant associated membrane structures. To date, eight different proteins participating directly in the biosynthetic pathway and its regulation are established (Guerriero et al., 2015). These are UDP-glucose pyrophosphorylase (UGPase), cellulose synthase, diguanylate cyclase, phosphodiesterase (PDE-A and PDE-B), and the recently discovered cellulose synthase operon genes *bcs*A, *bcs*B, *bcs*C, and *bcs*D (Basu et al., 2016). Each of these is a candidate target to obtain enhanced or reduced expression by genetic engineering. Genes *bcs*A, *bcs*B, *bcs*C, and *bcs*D appear to be translationally coupled and transcribed as a polycistronic mRNA with an initiation site 97 bases upstream of the coding region of the operon’s first gene. Gene complementation tests and gene disruption analyses reveal that these genes are essential in maximizing cellulose synthesis. The second gene (*bcs*B) encodes the cellulose synthase catalytic subunit. The structure of the first twenty-four amino acids of the sequence realized from this gene is remarkably similar to that of the signal peptides of the other bacterial secreted proteins, suggesting that cellulose synthase is being synthesized as a precursor and is deployed in the membrane after processing/activation. Although the exact functions of the other three gene products in cellulose synthesis are uncertain, it appears that they perhaps are important in the complex processes of cellulose transport and crystallization, or in regulating the synthase activity.

The microbial cellulose production and its responsible genetic organization have been studied in *Gluconacetobacter xylinum* (Ji et al., 2016)*.* The operon *bcs*ABCD contains four genes which are responsible for BC biosynthesis pathway, which was initially identified in *Acetobacter xylinus*. The BC synthase activity is guided by the first two genes of the operon *in vitro* (*bcs*A and *bcs*B), whereas the other two genes *bcs*C and *bcs*D are responsible for the transportation and packaging of glucan on the surface of the cell that leads to maximum BC production (Omadjela et al., 2013). There is another gene (*ccp*A) product which complements the BC synthesis *via* affecting the expression levels of *bcs*B, *bcs*C, and *bcs*A, which leads to maintaining the crystalline structure in cellulose ribbons (Nakai et al., 2013). Other bacteria such as *Agrobacterium tumefaciens* and *Rhizobium legunimosarum* contain the same operon (*bcs*A) that includes two genes (*bcs*A and *bcs*B). On the same loci of *bcs*A, there are two adjunct operons present (*cel*ABCD and *cel*DE). The gene *cel*E is mainly responsible for BC synthesis in *Agrobacterium tumefaciens* (Romling and Galperin, 2015), in addition to bacteria and four cellulose synthase genes (*ces*A) in *Phytophthora infestans*, an Oomycete fungus (Hua et al., 2015).

Catalysis of cellulose synthase appears to be rate-limiting. However, the level of UGPase, which is less specific to the process, may also be demonstrating rate limiting in certain (such as *Acetobacter*) strains. The permeability of glucose in poor media may be the slowest step, whereas the rate of gluconeogenesis may be the bottleneck when glycerol is used as the substrate. Thus, whether a particular factor is rate-limiting is also a function of a given set of physiological conditions.

The basic researchers and the bioinformaticians working cooperatively to generate data that would create *in silico* representations of microbial metabolism is the need of the hour. The models, when analyzed, would to lead bioinformatic and experimental study suggestions that would contribute to leads for more robust metabolism characterization for enhanced production. Opportunities in this technology may further enhance by delving deep into the cellulose-producing biogenetic pathway network. Furthermore, tailoring the cellulose structure at nano-level should be possible by genetically manipulating the cellulose-producing organisms.

## Applications of Microbial Cellulose

**FIGURE 2:**
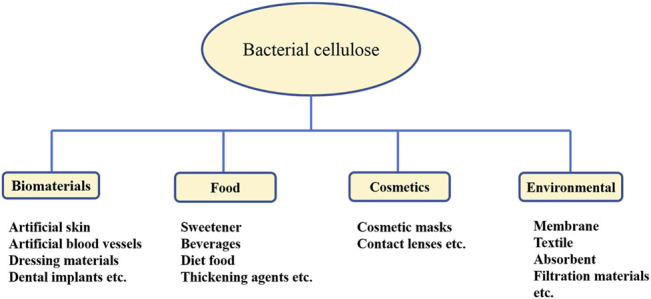
Categorized applications of BC in various sectors.

BC is made up of a highly crystalline form of cellulose without pectin, hemicellulose, and lignin. Being of versatile nature and bestowed with several unique properties, such as high water retention capacity, porosity, high degree of purity, ultrafine work, and stretchability, it is useful in various applications (Ullah et al., 2016). As BC is similar to plant cellulose in chemical structure but superior in properties, it is more useful in several applications such as medicine, drug delivery system, cosmetics, and food (Lin et al., 2013). The extensive use of cellulose in biomedical applications has been promoted since the last few decades; it shows the properties of a suitable scaffold base in promotion of cell and tissue growth due to its biocompatibility, biodegradability, and cost-effectiveness (O'Donnell et al., 2018). The various applications of BC are further detailed in the following text (Figure 2).

### Biomedical Applications

There are no macroscopic or hematological changes in mice even after repeated applications of BC (Moreas et al., 2016), which establishes their toxicity-neutrality. The major applications of BC are in the field of tissue grafting such as artificial skin, blood vessels, dressing materials in wound healing, and drug delivery agent in bone tissue. Due to the strong interface between hydroxyl groups, its crosslinking fibers show an attribute of self-assembly. The enhanced network of intra- and inter-hydrogen bonds provides a layer of high surface area and porosity that gives a novel site for drugs to bind inside the fibers (Li et al., 2011). It was reported that due to the nanomaterial attribute of BC, it provides better anchoring to the cells to adhere on the surface and proliferate rapidly in wound healing, bone grafting, and skin tissue engineering (Ul-Islam et al., 2013). It was reported in the early 90s for the first time that BC is helpful in rapidly replacing burnt skin tissues (de Oliveira et al., 2016; Moraes et al., 2016). Reportedly, BC provides an impermanent covering material to traumatic injuries, pressure sores, skin tears, diabetic wounds, and donor skin-graft sites (Ludwicka et al., 2013) due to its water permeability, elasticity, proper adherence to the wound sites, and as a physical check-point for harmful microbes. BC maximizes the healing rate, thereby helping in reducing pain at injury sites in several cases (Islam et al., 2021). Table 2 details few major applications of BC in the field of biomedical engineering.

**FIGURE 3:**
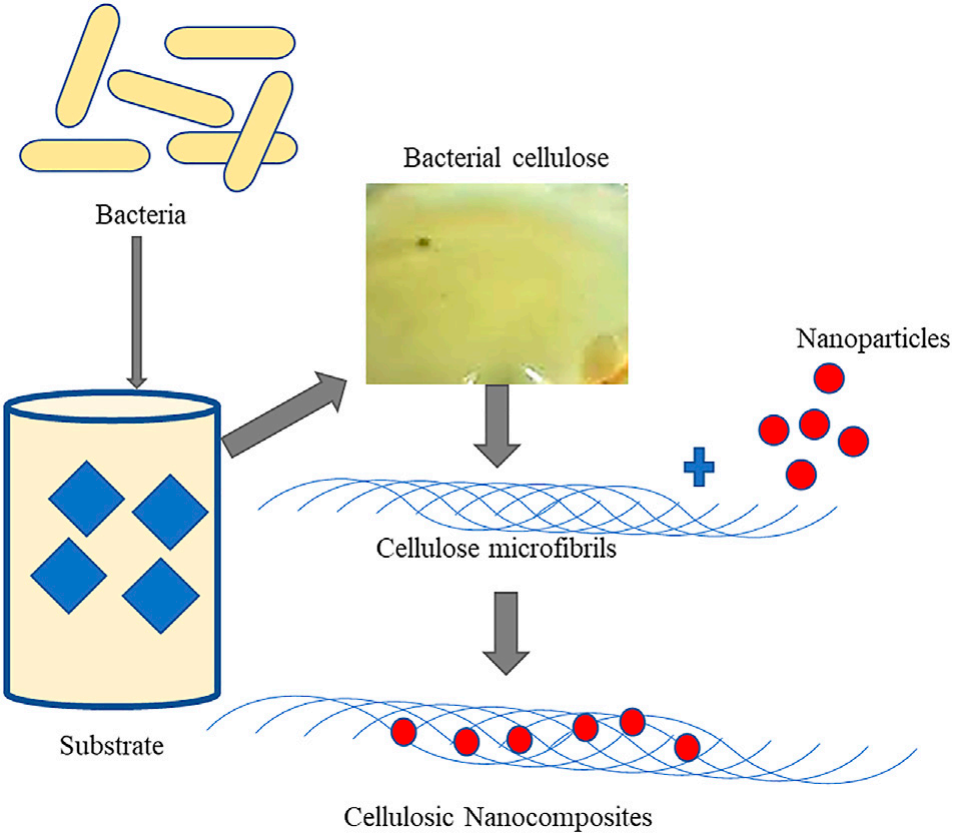
Graphical representation of synthesis of cellulose and its nanocomposites.

**FIGURE 4:**
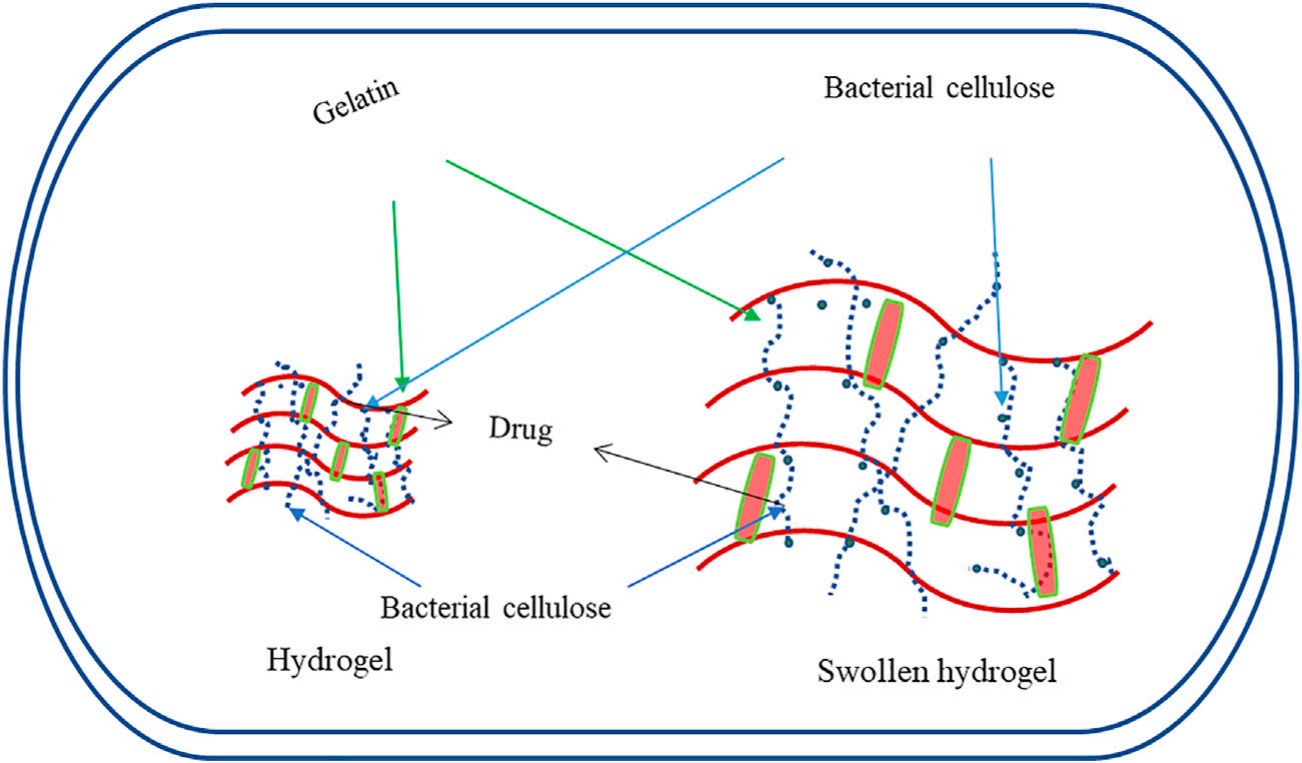
Graphical representation for the hydrogelling property of BC useful for nanoparticle-based drug delivery systems.

Other than the previously discussed direct applications of BC, improvising their properties are essential to enhance and broaden their applications. In this context, several nanocomposites have been developed with improvised properties (Figure 3). These nanocomposites are more thermostable and have better elasticity and tensility than native BC that collectively enhance the mechanical properties for application as BC-gelatine nanocomposites and BC-collagen (Albu et al., 2014; Moraes et al., 2016; Ilyas et al., 2019). Reports suggest that the BC-aloe gel–based composite formed by adding 30% (v/v) of aloe vera gel into the medium of BC has more water holding capacity and improved crystallinity than its native counterpart (Lazic and Falanga 2011) (Figure 4). BC-chitosan composite is another nanocomposite with slow water-releasing property useful in treating bed sore, ulcers, and other hard-to-heal wounds (Islam et al., 2017; Islam et al., 2020). Due to its biocompatibility nature, BC units are useful in bone healing as the human osteoblast cells responsible for healing of fractured bone exhibit a proclivity to adhere well to them.

As a source of drug delivery system, BC has been positive both in antimicrobial agents as well as in silver nanoparticle–based bacterial cellulose (Ag-BC) metallic biocomposites. Ag-BC is effective in antimicrobial activity on both Gram-positive as well as Gram-negative microorganisms (Lazic and Falanga 2011; Islam et al., 2021). BC has also shown positive results in the delivery of film-coated paracetamol, with the help of film-coating mechanism in *in vitro* studies (Halib et al., 2012).

**FIGURE 5:**
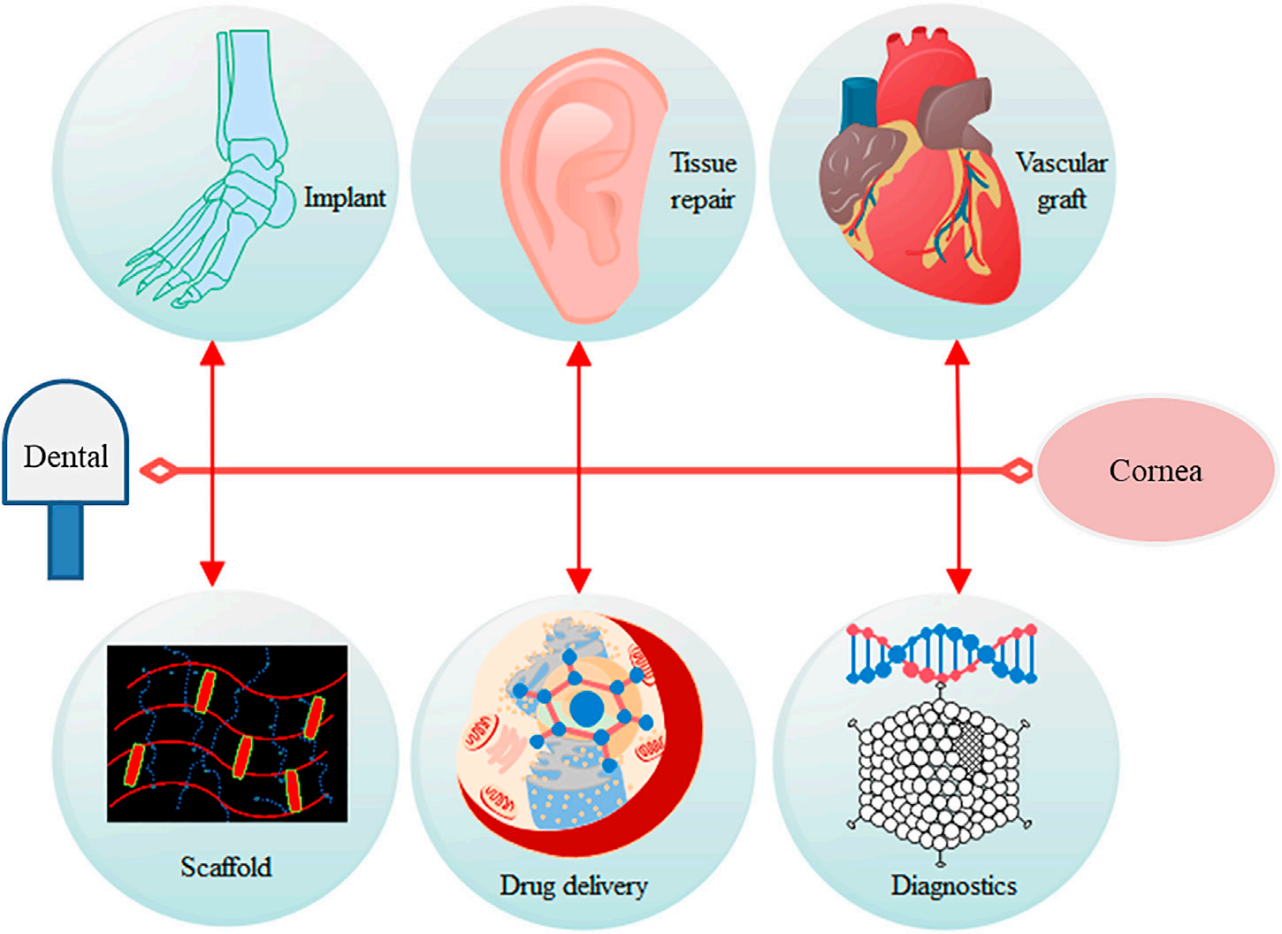
Possible wide applicability of bacterial cellulose in the biomedical field.

Several other applications of BC include dental implants, scaffold matrix, artificial cornea, tympanic membrane, and heart valve (Islam et al., 2021) (Figure 5). However, along with several properties and effectiveness of BC, there are some challenges regarding controlled release of drugs and control of pore size during synthesis and structural uniformity (composition similarity on surface as well as in the core). To make it a more value-added and cost-effective biomaterial, more research on these areas is warranted.

### Environmental Applications

BC can be used as a membrane filter for separation of various entities, namely, microorganisms, organic pollutants, and metal cations from contaminated water bodies. In a similar context, BC membranes were examined for removal of *E. coli* from a sanitary effluent. Alves et al. (2020) used the BC membrane to separate *E. coli* from dairy and textile effluents, and effectively removed the microbe even up to 10 cycles: the BC membrane as well as the BC membrane laminated with ß-chitin or deacetylated chitin sulfonate (S-DAC) for solute separation. The solute rejection of 85 and 95% was obtained for BC laminated with ß-chitin and BC laminated with S-DAC, respectively, while only 30% rejection could be obtained with normal BC membrane. In another report, acrylic acid–incorporated BC membrane could remove metal ions including heavy metals.

Additionally, nanocomposite materials made up of BC are successful as antimicrobial agents and in organic pollutant detection. The composite material based on BC and silver nanoparticle exhibited strong antimicrobial activity against *Staphylococcus aureus*, *Pseudomonas aeruginosa*, and *Escherichia coli* (Moreas et al., 2016). The resulting composite material (BC-Ag nanocomposite) that could release silver was found to be effective against *Staphylococcus aureus* for a period of 72 h. BC-associated nanocomposite has also been tested for the detection of organic analytes. For example, silver-incorporated BC nanocomposite was used to sense amino acids such as L-phenylalanine, L-glutamine, and L-histidine in aqueous solutions (Keshk and Gouda 2017). The composite material captured these organic molecules and results in surface-enhanced Raman scattering effect that was detected by Raman spectroscopy. BC hydrogels with gold nanoparticles were immersed in a solution containing 4-fluorobenzenethiol (4-FBT) and phenylacetic acid (PAA) in another investigation (Gupta and Diwan 2017). The hydrogels captured these organic analytes and underwent washing with DI water, followed by freeze drying or heat drying. The presence of (4-FBT) and PAA in the generated BC hydrogels was confirmed using Raman spectroscopy.

### Food Applications

Bacterial cellulose, as the name suggests, is produced by bacteria in a very pure form. BC produced in the media may contain the color and flavor of that media, for example, BC synthesized by bacteria on medium containing fruit syrup as substrate produced cellulose that contained flavor and pigment of the fruit (Shi et al., 2014). Likewise, the BC produced can be of various shapes, namely, filaments, sphere, films, particles, and whiskers (Shi et al., 2014). These characteristics of bacterial cellulose, such as high purity and the ability to change in flavor, color as well as various shapes and texture, makes it a potential candidate of food ingredient (Shi et al., 2014). The use of BC as a food ingredient improves food stability over a wide range of pH, temperature, and freeze-thaw conditions. Bacterial cellulose named Nata from the Philippines, due to its smooth mouthfeel and easy manufacturing process, is being used as traditional food dessert in South East Asia (Phisalaphong and Chiaoprakobkij 2012). The natural red pigment Monascus has been examined as the colorant for BC, and the resulting Monascus-BC complex that is stable in morphology and color gives a similar flavor to meat and acts as a meat replacement for vegetarian (Shi et al., 2014).

Other useful applications of BC in the food technology sector include thickening, gelling, and water binding (Han et al., 2018). When used as a thickening agent, BC is added to the ingredient paste, resulting in stickiness of the compositional material, which can be measured using a spoon (Han et al., 2018). Likewise, the application of BC as a stabilizing agent tends to retain the moisture content of the product, for example, application of BC in ice cream retains its shape for 60 min at least, whereas without BC, ice cream melts completely in a similar interval of time (Han et al., 2018). As a gelling agent, BC increases the texture and firmness of tofu (Han et al., 2018). BC when added to chocolate as a suspending agent entraps cocoa particles and the precipitation of cocoa particles can be avoided.

BC can be used to prepare low-fat food materials, for example, addition of BC in place of fat to Surimi increases the water-holding capacity due to the BC-enhanced network structure of Surimi (Lin et al., 2011). Likewise, adding 10% BC to meatball enhances the juicy, chew property of meatballs, making it a fat replacer in emulsified meat products (Khirrudin 2012). As per reports, BC when used as a dietary component can help in the reduction of body cholesterol (Ullah et al., 2016). The composite material made up of BC can be used as packaging material: the composite material made up of PLA (poly (lactic acid)) and BC was used as food packaging material. The composite material was reported to be showing better mechanical properties compared to pure PLA while still retaining its biocompatibility and transparency (Xiao et al., 2012). The antimicrobial agents can be incorporated into packaging materials to maintain food quality during storage. Shi et al. (2014) reported the use of nisin-incorporated BC as packaging material to control *Listeria monocytogenes* and total aerobic bacteria on the surface of vacuum-packaged frankfurters. Poly-l-lysine is also an effective antibacterial agent that can be incorporated with BC, and the resulting composite material not only retains the antibacterial activity but oxygen permeability through the composite is also reduced than general packing materials.

BC can be used as an immobilizing agent for enzymes and microbial cells. Recently, it has been reported that BC beads are used for immobilization of glucoamylase in the food industry. The BC gels of around 0.5–1.5 mm can enhance the activity and stability (at low temperature and pH values) of the enzyme (Wu et al., 2013). In a similar context, the fungal enzyme laccase was immobilized on the BC sponge through cross-linking with glutaraldehyde. The catalytic activity of crosslinked BC had observed over a wide range of pH, increased stability, and retained 69% of its original activity even after seven cycles (Chan and Chen, 2016). The use of enzymes such as horseradish peroxidase and laccases as biosensors *via* the immobilization method has also been reported (Lin et al., 2013). Likewise, the bacterial cell *Corynebacterium glutamicum* was immobilized on BC for the synthesis of L-lysine (Kirdponpattara and Phisalaphong, 2013).

### Other Miscellaneous

The main thought behind BC as a possible substitute for leather depends on the industrial production of cellulosic fiber by *Komagataeibacter* (Rebouillat and Pla, 2013). These microbes, alone or with other microbes, synthesize cellulose under aerobic condition, which in the form of aggregates/pellicles are accumulated in the extracellular medium. The cellulosic fibrils after drying and processing generate a tough leather-like substance with attributes that look like the animal-derived leathers that are being used in the shoe industry. A large amount of these pellicles could be generated *via* the industrially fermented simple nutrients. In spite of the interest shown in BC and other polymers by industries, academia (Moniri et al., 2017), and the art and fashion worlds (Kastner and Miltner, 2016), they have never been improved for use by the shoe industry.

Additionally, bacterial cellulose may be used as a leather substitute in clothing, decorative, and automobile industries. Certainly, factory-made leather potentials many recompense over animal leather. Like different real skins, BC sheets could be synthesized with straight edges, without scars, marks, and other defects; this could lead to reducing waste being a leather substitute.

## Future Prospects

Various laboratories involved in BC have developed bioproducts for industrial applications. Even if their specific engineering facts still need explanation, BC production is definitely scalable due to its relatively simple fermentation protocol. While studies to significantly reduce the production cost for BC through mass synthesis process have been conducted, its commercial production is yet far from reality. Furthermore, more biochemical and genetic studies are warranted to fully understand enhanced cellulose production.

The productivity of BC primarily defines its economic feasibility. The fermenter design must be suitable to contain the rapidly expanding culture while limiting mechanical disruption of the BC fibrillar matrix. A stirred tank reactor (STR) has the advantage of homogenizing the culture through agitation, resulting in a highly branched, 3-D reticulated structure. On the other hand, the high energy input for the mechanical power is its drawback. A normal lamellar cellulose pellicle with insignificant branching is generated in static (e.g., airlift bioreactor) culture. Although an airlift reactor has limited agitation power that might result in reduced broth fluidity, particularly at an elevated cellulose concentration, its energy cost is one-sixth compared to STR. Thus, the designing aspects of the fermenter such as the vessel shape and the impellers/blades to obtain the desirably modified fibrillar macroscopic nature of BC are necessitated. A design that could combine the benefits of an airlift as well as a STR or even a continuous cultivation (continuous mode) system could be useful. Using modified reactors such as a bioreactor with spin filter, a reactor with silicone membrane, rotating disk reactor, or even a rotary biofilm contactor could also be a practical strategy. However, a major obstacle in scaling-up such bioprocess is the accumulating metabolic byproducts that are wasteful and noxious, which need to be addressed. It appears that the full BC production potential, even by excellent producers like *G. xylinum* or *Acetobacter* sp., has not yet been realized, although other bacterial exopolysaccharides (Gullo et al., 2017) have been successfully produced at industrial-scale. This indicates that a greater and more obtainable production of such polymers from bacterial cells could be expected.

**FIGURE 6:**
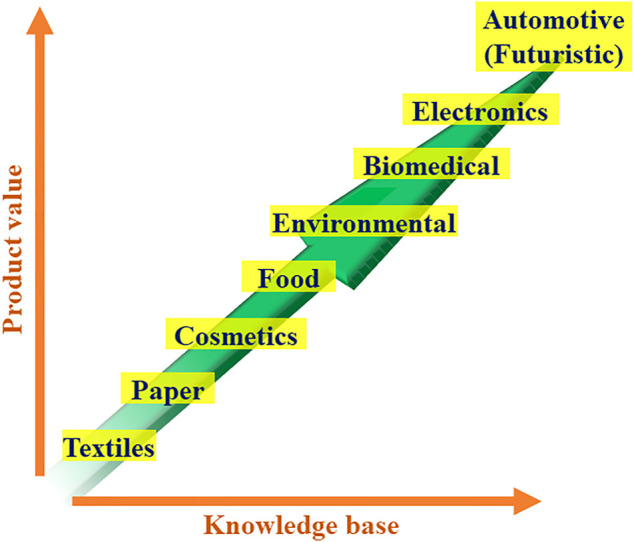
Envisioned future use of BC and BNC in high-value commercial products as the knowledge base grows.

The resulting crystalline, biodegradable, and ecofriendly nano-sized BCs are good candidate materials in the promising bio-nanotechnology in the major “green chemistry” commercial markets. Not underestimating some important steps that remain to be fundamentally and practically addressed, various laboratories have developed bioproducts of industrial reality with many possible applications. The basic and useful knowhow on nanocellulose materials thus fine-tuned could translate to high-performance nanostructures while promoting additional synergism. With a multi-disciplinary approach, the pilot scale and the demonstration plants can synergistically improve, optimize, and scale-up the laboratory-scale practices, and would help to engineer novel products having new functionalities. These would certainly favor the scheming of industrial-size performing reactors in appropriate environments and modern control equipment, and broaden the relevance base of BCs with applications in aramids and carbon fibers also used as reinforcing elements in automotive, military, aerospace and aeronautics, and marine and civil protection end-uses (Figure 6). Furthermore, merging such materials with bacterial nanocellulose (BNC) and establishing their values could be an area of significant interest.

Apart from being a strong and pure crystalline material, nanocellulose is a high modulus filler that improves modulus elasticity, tensile strength, and shear strength in fiber-reinforced polymers (FRPs); has a lower density than ceramic and/or metal fillers; an adhesive filler for lap shear strength; a good in-fiber filler for FRPs; and is useful in hierarchical composites (H-bonding hierarchical structure) and also in natural fiber coating with low coefficient of thermal expansion (CTE). It also has potential applications as an interface for natural fiber-reinforced composites, networked fiber structure, reinforcing sheets, filters, and supports. Although the natural fiber-reinforced composites could potentially be serious competitors to glass fiber composites, their high moisture absorption, thermostability, aggregation, interfacing, and enzyme degradability are few critical challenges that need to be addressed. In environmental biotechnology, mixed species environmental studies and biofilms thereto could greatly be advanced through the applications of BC. There is an abundance of Fe (III) oxide mineral, a good electron acceptor, which also provides a surface for electroactive biofilm to grow, both in the terrestrial (soils) and aquatic (sediments) environments. Being integral to the microbial consortia actively participating in decomposing organic matters and material cycling (including toxic metals), these biofilms are important. Such information on environmental consortia could motivate designing of synthetic electroactive microbial population and innovative bioenergy and bioremediation platforms to harvest energy from waste organics and immobilize contaminants (Mishra et al., 2018).

These new-age composite materials would require legislative support as well. Thus, along with the policy makers, the scientific, agricultural, and industrial communities need to come together to develop an alternative ecofriendly composite material that is cheaper and more suitable for structural applications.

## Conclusion

Cellulose nano(bio)composites production is still in its infancy as their behavioral understanding is limited due to the complex molecular nature. Thus, it necessitates an enhanced understanding on the diffusion and dissolution of the nanocellulose fibrillar network. The potentials of BC and BNC in the world of new-age materials with excellent performance are huge. The potential of cellulose nano-reinforcement can then be entirely realized with a better understanding in these. Some challenges to address in extending cellulose nanocomposite applications are related to the lack of appropriate low-energy scale-up technologies and its related high cost, and its isolation and agglomeration.

In spite of the huge potential applications of BC-BNC, the mechanism of cellulose biosynthesis remains poorly understood. Gene-level engineering for cellulose production ability may remain a challenge as it is difficult to transfer across organisms. Bacterial BNC production including BNC production kinetics, pathogenicity, economics, and product quality/consistency may need to be addressed for scaled-up applications. A few of the strategies could be alternate feedstock, genetic engineering, process engineering, low cost scaled-up production, isolation methods, compatibilization chemistries, and composite processing. Biocomposites are potentially similar to natural fiber composites with additional advantages of biodegradability of both the matrix and the fiber. It calls for extensive research efforts for its wider applications, product ranges, and deeper and meaningful market presence. Future research on BC and BNC may focus to overcome existing green, value-added product demands. Although there are noteworthy industrial/commercial scale applications of BC and BNC, still more (such as use of BC in Philippine nata) needs to be realized. BC is an attractive high-performance natural nanomaterial that can complement plant cellulose in biomanufacturing operations. Realizing such opportunities to reduce the production cost and progressive applications of biocomposites would need public and private investment.

## Abbreviations

Ag-BC, silver nanoparticle-based bacterial cellulose; BC, bacterial cellulose; bcs, cellulose synthase operon genes; CTE, coefficient of thermal expansion; FTIR, Fourier-transform infrared spectroscopy; GDPG, guanidine diphosphoglucose; G. xylinus, *Gluconacetobacter xylinus*; STR, stirred tank reactor; FRPs, fiber-reinforced polymers; PDE, phosphodiesterase; UDPG, uridine diphosphoglucose; UGPase, UDP-glucose pyrophosphorylase.
